# Price promotions on healthier compared with less healthy foods: a hierarchical regression analysis of the impact on sales and social patterning of responses to promotions in Great Britain^1^,^2^,^3^,^4^,^5^

**DOI:** 10.3945/ajcn.114.094227

**Published:** 2015-02-11

**Authors:** Ryota Nakamura, Marc Suhrcke, Susan A Jebb, Rachel Pechey, Eva Almiron-Roig, Theresa M Marteau

**Affiliations:** 1 From the Behaviour and Health Research Unit, University of Cambridge, Cambridge, United Kingdom (RN, MS, SAJ, RP, and TMM); the Health Economics Group, Norwich Medical School, University of East Anglia, Norwich, United Kingdom (RN and MS); the Centre for Health Economics, University of York, York, United Kingdom (RN and MS); the UKCRC Centre for Diet and Activity Research, Cambridge, United Kingdom (RN and MS); the Nuffield Department of Primary Care Health Sciences, University of Oxford, Oxford, United Kingdom (SAJ); and the Medical Research Council Human Nutrition Research, Elsie Widdowson Laboratory, Cambridge, United Kingdom (EA-R).

**Keywords:** food purchasing, Great Britain, price promotion, public health, public policy

## Abstract

**Background:** There is a growing concern, but limited evidence, that price promotions contribute to a poor diet and the social patterning of diet-related disease.

**Objective:** We examined the following questions: *1*) Are less-healthy foods more likely to be promoted than healthier foods? *2*) Are consumers more responsive to promotions on less-healthy products? *3*) Are there socioeconomic differences in food purchases in response to price promotions?

**Design:** With the use of hierarchical regression, we analyzed data on purchases of 11,323 products within 135 food and beverage categories from 26,986 households in Great Britain during 2010. Major supermarkets operated the same price promotions in all branches. The number of stores that offered price promotions on each product for each week was used to measure the frequency of price promotions. We assessed the healthiness of each product by using a nutrient profiling (NP) model.

**Results:** A total of 6788 products (60%) were in healthier categories and 4535 products (40%) were in less-healthy categories. There was no significant gap in the frequency of promotion by the healthiness of products neither within nor between categories. However, after we controlled for the reference price, price discount rate, and brand-specific effects, the sales uplift arising from price promotions was larger in less-healthy than in healthier categories; a 1-SD point increase in the category mean NP score, implying the category becomes less healthy, was associated with an additional 7.7–percentage point increase in sales (from 27.3% to 35.0%; *P* < 0.01). The magnitude of the sales uplift from promotions was larger for higher–socioeconomic status (SES) groups than for lower ones (34.6% for the high-SES group, 28.1% for the middle-SES group, and 23.1% for the low-SES group). Finally, there was no significant SES gap in the absolute volume of purchases of less-healthy foods made on promotion.

**Conclusion:** Attempts to limit promotions on less-healthy foods could improve the population diet but would be unlikely to reduce health inequalities arising from poorer diets in low-socioeconomic groups.

## INTRODUCTION

Price promotions are commonly used in store with the aims of boosting purchasing by reducing the price of products as well as possibly stimulating impulsive purchases by increasing the prominence of items in store (e.g., via tags and placement). There is a growing concern that such promotional activities by the food industry may contribute to poor dietary intake particularly in individuals who are more socially deprived (1–3). It was also suggested that price promotions on less-healthy products might lure consumers away from healthier, higher-priced options and that the industry has disproportionately promoted less-healthy but more-profitable options (4). If so, there might be a case for public policy to regulate the promotional activities of industries to help achieve, or at least not hamper, public health nutrition goals.

However, there is a paucity of empirical evidence available in the public domain, and the existing claims about a bias in the use of price promotions toward less-healthy items largely rest on anecdotal reports. Although the general responsiveness of consumers to price promotions received substantial attention in the marketing literature (5, 6), and there is a fast-growing body of research on the effect of price per se on healthier compared with less-healthy purchasing or consumption (7–13), there has been relatively little research to consider whether consumer uptake of promoted products differs for healthier or less-healthy products. Moreover, it is unclear whether the impact of promotions varies by the socioeconomic characteristics of consumers. In this study, we sought to fill this evidence gap. We examined whether promotions on less-healthy products increased sales more than promotions on healthier products by using data from supermarkets across the United Kingdom.

We also sought to explore whether social disparities in the healthiness of food purchased were attributable to differences in responses to retail price promotions. In an earlier study, by using the same survey data, we showed significant socioeconomic patterning in the healthiness of food purchases (14). However, the mechanisms that could account for such patterns remain underexplored. In the current study, we tested one potential mechanism, i.e., price promotions; we investigated the differential use of price promotions across socioeconomic groups to explore whether this may be one contributor to diet-related health inequalities (15).

We designed our study to address the following 3 questions: *1*) Are less-healthy foods more likely to be promoted than healthier foods? *2*) Are consumers more responsive to promotions on less-healthy products than promotions on healthier ones? *3*) Are there socioeconomic differences in food purchases in response to price promotions?

## METHODS

### Data

We used a secondary data source, the Kantar WorldPanel survey (14, 16), which includes purchase records of 26,986 households in the United Kingdom throughout 2010. Households were recruited by a data company (Kantar WorldPanel), and the authors were not involved in the data collection. Data-collection procedure was as follows: the United Kingdom Office of National Statistics census information and the United Kingdom Broadcasters’ Audience Research Board Establishment survey were used to determine quotas for recruitment. The data company purchased potential participant lists from another company and recruited participants by sending postal mails and e-mails. Recruited participants received vouchers for high-street retailers and/or vouchers for leisure activities [in total for an average monetary equivalent of £100 (∼$160) per household per year]. Recruited households were nationally representative in terms of region, age group, and household size.

The survey includes purchase records of all foods and beverages that were taken home from supermarkets and similar stores in 2010. Sampled households were asked by the data company to record all purchases using barcode scanners and to send digital images of cash-register receipts to the company. The data contain rich information on purchases, including the price at which they were bought, whether they were bought on promotion, the number of packets purchased, and the retail chain from which the product (Stock Keeping Unit) was purchased. The data also include detailed information on product characteristics, including information on the brand, manufacturing company, and nutritional content.

We constructed a cross-sectional data set of 11,323 individual products in 135 food and drink categories, which were purchased by panel households in leading United Kingdom supermarket chains [i.e., the “main parties” as defined by the United Kingdom Competition Commission (17)]. The food and drink categories reflected those used in the retailing sector (Kantar WorldPanel; see **Supplemental Table 1** for more information). With the use of transaction records in the data, we calculated the total number of units of each product sold to panel households across the country over 52 wk. In keeping with common practice in the related literature (13, 18–20), we restricted the set of products to the more-popular items; in particular, we included only products that were purchased at least once in each of the 52 wk by any of the panel households, irrespective of whether the products were on promotion or not.

### Frequency of promotions

To assess consumer responses to price promotions, it is essential to measure the number of price promotions available for each product. However, in commonly available data sets (including ours), a price promotion is recorded for a given item in a given store only if that item has been purchased on promotion from that store by panel households. This purchase-based nature of existing data sets has thus far prevented researchers from measuring the frequency of price promotions at population level.

In the absence of a directly observable measure in the data, we estimated the frequency of promotions for each product by exploiting a particular feature of the retail policy in major United Kingdom grocery retailers. We focused on the following 11 “main parties” of United Kingdom multiple grocers: Tesco (sales market share in 2010 was 24%), Asda (sales market share in 2010 was 12.8%), Sainsbury (sales market share in 2010 was 12.5%), Morrisons (sales market share in 2010 was 9.8%), Waitrose (sales market share in 2010 was 3.1%), Iceland (sales market share in 2010 was 1.7%), Lidl (sales market share in 2010 was 1.6%), Aldi (sales market share in 2010 was 1.5%), M&S (sales market share in 2010 was 1.3%), Netto (sales market share in 2010 was <1%), and Budgens (market share information not available), as defined by the United Kingdom Competition Commission (17). The total market share of these grocers in 2010 was ∼70% (21). The Commission confirmed that the stores followed a national pricing policy according to which stores operated the same pricing (and, thus, the same price promotions) in all branches. This institutional feature provided us with the opportunity to estimate the number of promotions run in the country in a given time period; if we observed any transaction involving a product on promotion in a given store, we could assume that the product was also on price promotion in the other branches of the same supermarket chain.

The frequency of promotions for each product was defined by the number of branches (22) that ran a promotion on the product in a given week aggregated across the 11 supermarket chains and 52 wk. Each branch could run a promotion on a given product (Stock Keeping Unit) only once at a given point in time, and hence, the number of branches that ran a promotion on a product gave the number of promotions on the same product. See **Supplemental Data section 2** for additional details.

### Product and category healthiness

The nutrient profiling (NP) model developed by the United Kingdom Food Standards Agency was used to capture the healthiness of products (23). This method assigns a score for each food calculated from the energy density, saturated fat, sugar, sodium, fiber, and protein contents together with an estimate of the fruit, vegetable, and nut contents, thereby providing a unified measure of healthiness across all available food and drink products. The NP model applies to all food and drink products equally without exemptions or category-specific criteria (23). However, the definition of healthier and less-healthy products typically uses different cut points for foods and beverages, reflecting the very different energy densities of the 2 groups, and we adopted this convention. Note that, as the NP score increases, the healthiness of the product declines. Compared with other NP models, this NP score was shown to perform well when matched to a standard ranking of foods by >700 nutrition professionals (24). Intake of high-scoring foods was shown to act as a risk factor for obesity (25). Category-level healthiness was calculated by taking the mean NP score for products within the category.

### Analytic framework

#### Are less-healthy foods more likely to be promoted than healthier foods?

To address this question, we set up a product-level regression model of sales and assessed the relation between the frequency of promotions and NP score. In supermarkets, each product was nested by product category (135 categories in our data), and hence, our product-level data set had a 2-level structure (i.e., between-category and within-category variations in healthiness). First, we estimated the association between the frequency of promotions and NP score of various food categories (i.e., between-category differences). Next, we estimated the relation between promotions and the NP score at the product-level separately by the food category (i.e., within-category differences). These 2 step estimations were conducted simultaneously via a hierarchical regression approach (26). For item *j* in category *c*, the following base model was specified:


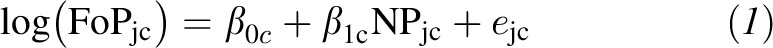


FoP_jc_ refers to the frequency of promotion of item *j* in category *c* and NP represents the nutrient profile. The term *e*_jc_ is the idiosyncratic error. This basic estimation was used to tell whether less-healthy items were more frequently promoted than healthier ones. We further specified that the baseline frequency of promotions (intercept: β_0c_) and the association with the NP score (slope: β_1c_) varied by dietary category, and this variation was a function of the genuine healthiness of each dietary category


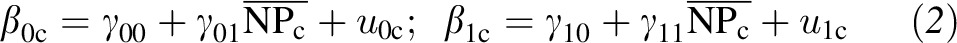



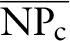
 is the mean NP score of products in category *c*. Now, the model could distinguish between product-level (i.e., within-category) effects and category-level (i.e., between-category) effects of healthiness, which were estimated separately in the regression analysis. See **Supplemental Data section 3** for full technical details.

#### Are consumers more responsive to promotions on less-healthy products?

To address this question, we investigated differential effects of the frequency of promotions on product sales by the NP score of products. The analysis assessed whether price promotions increased sales of less-healthy compared with healthier foods (between-category effect). The analysis also addressed whether sales of less-healthy versions within a given food category increased more in response to promotions than did healthier versions in the same food category (within-category effect). Again a similar hierarchical regression approach was used. The baseline product-level purchases equation is given by


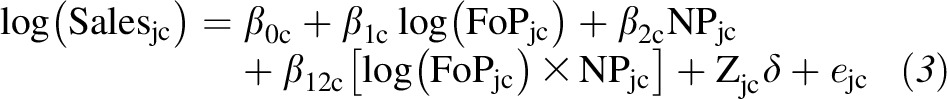


The outcome variable was the log of total number of products *j* in category *c* that were purchased by the panel households over 52 wk. The interaction term [log(FoP_jc_)×NP_jc_] was used to measure whether and, if so, to what extent the effect of promotions varied by the healthiness of the product. The vector *Z*_jc_ included a set of product-level covariates known to affect sales, including the reference price, average rate of price discount when promoted, and a set of indicators of brands (which captured the brand-specific features of each product).

Similar to the previous analysis, category-specific coefficients were modeled as follows:


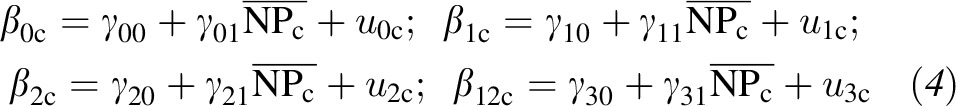


The model nested the within-category and between-category sales effects of promotion by healthiness, which, again, were estimated separately in the regression analysis. All models were estimated via a restricted maximum likelihood technique (27). See Supplemental Data section 3 for full technical details.

#### Are there socioeconomic differences in food purchases in response to price promotions?

To address this question, we constructed 3 subsamples that focused on purchases that were made by *1*) high–socioeconomic status (SES) households, *2*) middle-SES households, and *3*) low-SES households and repeated the previous analysis (on the basis of NP scores) for each group. The SES of the household was defined by the occupation of the household head using the United Kingdom Registrar General’s classification [high: higher managerial and professional; middle: white collar and skilled manual; and low: semiskilled and unskilled manual (28, 29)]. Other socioeconomic indicators such as household income and education were not used because of a substantial number of nonresponses. Observations with missing information (such as the NP score) were excluded from the analysis (6 cases, and no imputation was made for missing variables). Stata MP Version 12 software (StataCorp) was used for all analyses.

## RESULTS

**Table 1** shows characteristics of participating households (main shoppers) by socioeconomic groups. The total number of households was 26,986 with 5667 households in the high-SES groups, 14,870 households in the middle-SES group, and 6449 households in the low-SES group. There were gradients in household income, education level of the main shopper, and BMI. Although all households were included to calculate product sales, note that there were substantial item nonresponses in the information on the above characteristics (household income, education, and BMI).

**TABLE 1:** Household (main shoppers’) baseline characteristics by socioeconomic group^1^

	All	*n*	High-SES^2^ group	*n*	Middle-SES group	*n*	Low-SES group	*n*
Age, y	48.60 ± 15.84^3^	26,986	47.59 ± 15.20	5667	47.83 ± 15.77	14,870	51.25 ± 16.27	6449
Age groups (y), %		26,986		5667		14,870		6449
≤29	11.9		10.3		12.7		11.1	
30–44	36.0		41.4		37.3		28.4	
45–59	26.5		25.9		26.0		28.1	
≥60	27.7		24.4		26.0		34.3	
Sex (F), %	78.9	26,986	75.5	5667	79.7	14,870	80.3	6449
Ethnicity (whites), %	95.2	25,473	93.3	5429	95.2	14,008	96.8	6036
Household income,^4^ £	172,621 ± 11,002.5	20,474	25,123.1 ± 12,317.1	4367	17,271.8 ± 9844.2	11,299	10,099.2 ± 6523.7	4808
Age finished education (y), %		25,369		5469		13,952		5948
0–15	18.6		8.6		17.3		30.8	
16–18	42.4		30.6		45.6		45.8	
≥19	37.9		59.8		35.8		22.6	
Currently in education, %	1.1		1.0		1.3		0.8	
BMI, kg/m^2^	27.39 ± 5.89	12,008	26.62 ± 5.26	2898	27.27 ± 5.81	6563	28.57 ± 6.58	2547
Country of residence, %		26,986		5667		14,870		6449
England	86.3		87.3		86.5		85.1	
Scotland	8.6		7.9		8.5		9.1	
Wales	5.1		4.7		5.0		5.8	
Total households, *n*	26,986		5667		14,870		6449	

1 Data are from the Kantar WorldPanel Survey 2010. Households: *n* = 26,986. There were substantial numbers of item nonresponses in the following variables: ethnicity (1513 cases), household income (6512 cases), education (1617 cases), and BMI (14,978 cases). Therefore, the information for these variables is for reference only.

2 SES, socioeconomic status.

3 Mean ± SD (all such values).

4 Household income was adjusted for household size and composition.

The mean (±SD) NP score for food products at a product-category level was 4.54 ± 6.96 and ranged from the healthiest at approximately −10 (fruit and vegetables) to the least healthy at ∼22 (butter, margarine, and chocolate confectionery). At the individual product level, the mean was 3.72 ± 9.17. The mean frequency of promotions (i.e., number of branches that ran a promotion on a product in a given week) for each product was 481.4 ± 735.8 branches/wk.

**Table 2** presents descriptive statistics for the number of packs of each product purchased per 1000 households in 2010 separately by NP score and sales made on and off promotion. In this table, food categories that scored ≥4 in the category mean NP score, and beverages that scored one or higher were classified as less-healthy categories and, otherwise, as healthier ones. Products that scored above the median NP score within each category were classified as less-healthy versions and, otherwise, as healthier ones. In total, 6788 products were in healthier categories, and 4535 products were in less-healthy categories. Results were qualitatively similar when a different cutoff of healthiness was used (see **Supplemental Table 2** for sensitivity checks).

**TABLE 2:** Number of packs purchased on and off promotion per product per 1000 households by nutrient profiling score^1^

	Healthier category (*n* = 78 food/beverage categories)^2^				Less-healthy category (*n* = 57 food/beverage categories)			
	Healthier version		Less-healthy version		Healthier version		Less-healthy version	
	On promotion	Off promotion	On promotion	Off promotion	On promotion	Off promotion	On promotion	Off promotion
Profiling score, *n*	−3.2 ± 3.4^3^		1.6 ± 5.5		7.3 ± 7.7		16.9 ± 6.3	
Branches running price promotion per product per week, *n*	446.9 ± 714.1		536.2 ± 823.8		477.7 ± 726.8		492.9 ± 670.5	
Packs purchased per product per 1000 households, *n*								
All households	30.8 ± 111.9	46.1 ± 107.2	34.3 ± 104.6	67.1 ± 262.5	18.9 ± 47.0	29.0 ± 38.0	21.8 ± 57.3	30.0 ± 38.7
High SES households (*n* = 5667)^4^	33.3 ± 138.0*	47.9 ± 110.8*	33.6 ± 97.0*	65.5 ± 247.6*	18.2 ± 45.7*	27.7 ± 34.9	20.7 ± 58.4*	28.3 ± 40.8
Middle SES households (*n* = 14,870)^4^	31.6 ± 118.1*	46.6 ± 116.2*	34.3 ± 110.6*	68.0 ± 274.6*	19.1 ± 48.9*	28.8 ± 39.7*	21.9 ± 57.8*	29.8 ± 37.0
Low SES households (*n* = 6449)^4^	26.1 ± 92.2	42.7 ± 102.4	34.0 ± 120.1*	65.5 ± 273.0*	18.1 ± 57.6*	29.6 ± 44.8*	21.7 ± 63.3*	31.3 ± 47.7
Products included in the analysis, *n*	4316		2472		2560		1975	

1 Data are from the Kantar WorldPanel Survey 2010. Households: *n* = 26,986; total products: *n* = 11,323. SES, socioeconomic status.

2 Food categories that scored ≥4 nutrient profiling score and beverages that scored ≥1 were grouped in the less-healthy category and, otherwise, in the healthier category.

3 Mean ± SD (all such values).

4 Within each column, SES differences in the purchasing outcome were tested. Mean numbers that do not share an asterisk (*) were significantly different from each other at the 5% level on the basis of 2-sided *t* tests. Tests were Bonferroni corrected.

As for healthier food categories, Table 2 suggests that higher-SES groups bought more products from healthier versions of healthier food categories than did lower-SES groups for purchases made both off and on promotion. In terms of less-healthy food categories, socioeconomic differences were predominantly shown in off-promotion sales; the sales of less-healthy foods off promotion were significantly greater for the lower-SES group than for the highest-SES group.

### Frequency of promotions by NP score

**FIGURE 1:**
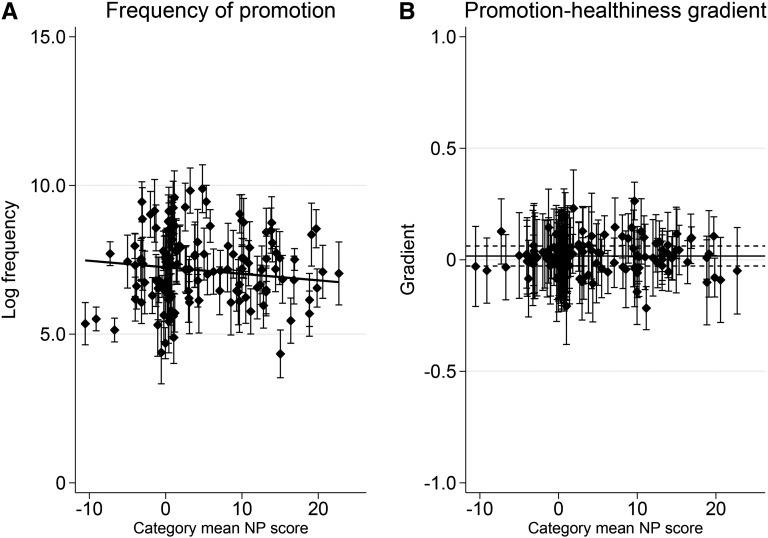
Empirical Bayes predictions of the log frequency of price promotion for individual categories, i.e., the between-category effect (A), and empirical Bayes predictions of the association between promotions and NP score within each category, i.e., the within-category effect (B). Effects represented were derived from results of the hierarchical regression analysis (see Supplemental Data section 3 for technical details and Supplemental Table 3 for complete regression results). For both panels A and B, 95% CIs of predictions are presented. The coefficient of the slope in panel A was −0.022 (*P* = 0.272; *z* test; *n* = 11,323; Supplemental Table 3). A positive gradient in panel B meant that promotions were more frequent in less-healthy than in healthier versions of foods within the category. The horizontal line and associated dashed lines show the overall size of effects with 95% CIs (0.0168; *P* = 0.462; *z* test; *n* = 11,323). NP, nutrient profiling.

**Figure 1** summarizes the estimation results of the hierarchical model (see **Supplemental Table 3** for complete results and **Supplemental Table 4** for sensitivity checks).

Figure 1A illustrates the estimated frequency of promotions by food category and shows that the frequency of promotions varied substantially across categories. The estimated mean of the log frequency was 7.25 (i.e., 1405.3 branches running promotions per product per week), with an SD across categories of 1.40 (Supplemental Table 3). The straight line in the graph shows the overall relation between promotions and the category-level NP score (i.e., the between-category relation). The slope coefficient was −0.022 (*P* = 0.272), which was small and statistically indistinguishable from zero, implying that promotions were equally likely in healthier and less-healthy food categories.

Figure 1B shows the relation between NP score and promotions within each category (i.e., the within-category relation). Gradients representing the association between frequency of promotions and NP scores within each category were plotted against the mean NP score of the category. A positive gradient implied that promotions were more frequent in less-healthy than in healthier versions within a given category. The horizontal line and associated dotted lines show the overall (average) gradient, which was 0.0165 (*P* = 0.462) and insignificant. Therefore, by looking at the within–food category variation, promotions were overall equally likely on healthier and less-healthy versions of the foods. At the individual category level, gradients were generally small and insignificant. However, there were a few cases in which price promotions were skewed toward less-healthy versions (e.g., cakes, cheese, and sauces; **Supplemental Table 5**).

The overall result was also replicated when applied to the following 2 specific types of promotion separately: simple price reductions and multibuys (e.g., “buy-one-get-one-free” and “X for $Y” (**Supplemental Tables 6** and **7**). However, promotions on less-healthy versions of foods were characterized by a bigger discount rate than were those for healthier foods (gradient: 0.00163; *P* = 0.058; **Supplemental Table 8**).

### Differential consumer responses to promotions by NP score

**FIGURE 2:**
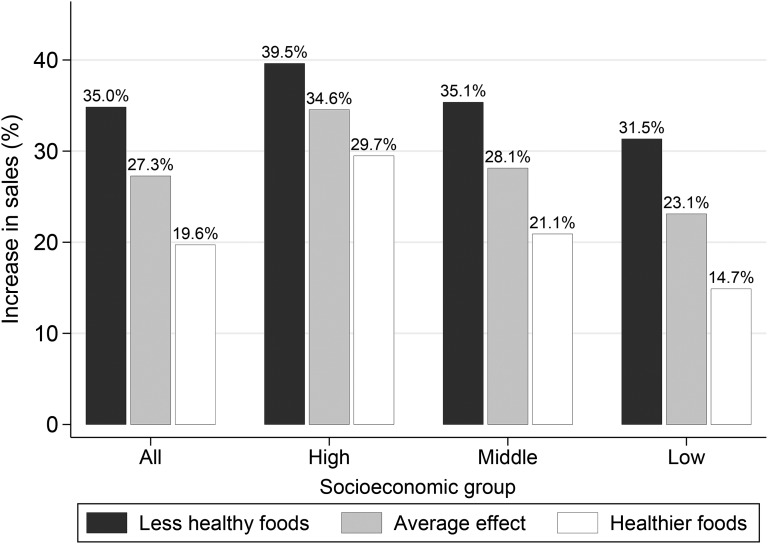
Effects of price promotions on sales by category-level NP score and socioeconomic group. Effects represented were predicted from the hierarchical regression analysis (see the regression model in the Analytic framework section and Supplemental Table 9). The gray bar shows the average percentage increase in sales when the frequency of promotions was raised by 10% [the bar corresponds to 10 times the coefficient of log(FoP)] presented separately by socioeconomic groups. Black and white bars show effects on less-healthy and healthier food categories, respectively, in which the category-level NP score was greater or smaller, respectively, than the mean by 1 SD point, whereas other factors remained fixed. The effect size corresponds to the coefficient of log(FoP)×NP) multiplied by the SD. The figure shows the between-category effect only. Within-category effects were indistinguishable from zero for all groups (Supplemental Table 9) and, therefore, are not visualized. See Supplemental Data sections 3 and 6 for additional technical details. FoP, frequency of promotion; NP, nutrient profiling.

**Figure 2** summarizes key results of the regression analysis regarding the association between unit sales and the frequency of promotions by NP score (see **Supplemental Table 9** and **Supplemental Figure 1** for complete results and additional technical details).

A 10% increase in the frequency of promotions was associated with an increase in sales of 27.3% (95% CI: 20.6%, 33.9%; *P* < 0.01) for the whole population (average effect). The sales uplift from price promotions was significantly larger for less-healthy than for healthier food categories. An SD point increase (6.96 points) in the category mean NP score (implying that the food category became less healthy) was associated with, all else being equal, an additional 7.7–percentage point increase in sales (*P* < 0.01; Supplemental Table 9) (i.e., the overall effect increased from 27.3% to 35.0%).

The sales uplift was also shown within each SES group. However, the magnitude of sales uplift was greater in higher- than for lower-SES groups for both healthier and less-healthy food categories (Supplemental Table 9 and **Supplemental Table 10**). Moreover, SES differences in the sales uplift were more marked in healthier than in less-healthy food categories; for less-healthy food categories, the sales uplift for high-, middle-, and low-SES group was 39.5%, 35.1%, and 31.5%, respectively, whereas in healthier food categories, it was 29.7%, 21.1%, and 14.7%, respectively.

By contrast, within a given category, the NP score of the product did not uniformly or significantly moderate the effect of promotions, although for some categories, a moderation effect did exist (see Supplemental Table 9 and **Supplemental Table 11** for separate regressions by product category).

### Price elasticity

Effects of the reference price (or nonpromotional price) and price discount associated with a price promotion were also estimated as control variables (Supplemental Table 9). The elasticity of the reference price within category was −0.64 (95% CI: −0.67, −0.61; *P* < 0.01), which implied that a 1% increase in the reference price led to a decrease in sales by 0.64% within a given category. The elasticity was larger for lower- than for higher-SES groups; the elasticity equaled −0.47 (95% CI: −0.51, −0.43; *P* < 0.01) for the high-SES group, −0.63 (95% CI: −0.66, −0.60; *P* < 0.01) for the middle-SES group, and −0.82 (95% CI: −0.86, −0.78; *P* < 0.01) for the low-SES group. The within-category elasticity of the price discount was 1.44 (95% CI: 1.32, 1.55, *P* < 0.01); a 1% increase in the depth of price discount led to a sales uplift by 1.44% within a given category. The effect was similar in size across SES groups, whereby it was 1.44 (95% CI: 1.31, 1.57; *P* < 0.01) for the high-SES group, 1.44 (95% CI: 1.32, 1.56; *P* < 0.01) for the middle-SES group, and 1.43 (95% CI: 1.29, 1.58; *P* < 0.01) for the low-SES group. Our results for the price elasticity between categories were nonsignificant for both the reference price and price discount.

## DISCUSSION

Despite earlier anecdotal evidence to the contrary, we showed that, overall, less-healthy items were no more frequently promoted than were healthier ones. However, after controlling for the price, price discount, and brand-specific effect, the sales uplift associated with price promotions was larger in less-healthy than in healthier food categories, which confirmed our main hypothesis. Products from less-healthy food categories are often nonperishable, whereas those from healthier food categories (in particular fruit and vegetables) are perishable. Therefore, stockpiling during a promotion may be more likely to happen for less-healthy food categories, which could explain the finding.

Higher-SES groups were more responsive than lower SES groups to promotions for both healthier and less-healthy foods. The reasons for this could not be determined from these data but may have been because the ability to respond to promotions is a function of shopping-related cognitive abilities, information, and skills [all of which have been shown to correlate with SES (30)] rather than the need to make monetary savings (31). In addition, making the most effective use of promotions may involve stockpiling items while they are on promotion, thereby requiring financial and spatial resources, which may also have contributed to the observed social patterning in the use of promotions.

These SES differences in the responsiveness to promotions were more pronounced in healthier than in less-healthy categories (Figure 2). Table 2 also revealed that there was a significant SES gap in the sales of healthier foods on promotion, whereas there was no such gap in the sales of less-healthy foods on promotion. These results suggested that the socioeconomic gap in the on-promotion sales was driven by differences in purchases of healthier rather than less-healthy foods.

There was also an SES gap in the sales of both healthier and less-healthy foods that were made off promotion (Table 2). This result was broadly in line with the SES patterning in terms of overall purchasing shown in the previous literature (14, 32, 33). Hence, SES differences in the larger proportion of off-promotion sales laid the foundation of the SES gap in food purchasing, which was exacerbated by promotional activities. Furthermore, elasticities of both the reference price and price discount were larger for the low- than for the high-SES group.

### Strengths and limitations

To our knowledge, the current study provides the first population-level quantitative assessment of the relation between the frequency of price promotions and healthiness of food purchases in the main supermarket chains in the United Kingdom. The analysis involved a considerably larger sample size than in existing studies on price promotions. Our study focused on temporary price changes that often augment the prominence of items in the store through tags and placement. Hence, the research usefully complemented existing studies on the role of price in healthy food purchasing more generally, which have had implications mainly for taxation or subsidization (i.e., permanent price changes).

To our knowledge, we also provided the first systematic assessment of a channel through which social disparity of food purchases and intake may occur. Although we and others previously showed social patterning of diet quality (proxied, for instance, by the proportion of less-healthy foods in total intake of energy) (14, 32–34), the underlying mechanisms as well as potential policy implications have rarely been tested, except for studies on food price (35–40). Although we had hypothesized that price promotions on less-healthy foods may be a plausible mechanism, our findings led us to reject this hypothesis.

In interpreting the findings, several limitations need to be borne in mind. First, our measure of the frequency of promotions was inevitably limited. The construction of the variable relied on the national pricing policy operated in leading United Kingdom supermarkets. However, the policy is known to be imperfectly adhered to in places characterized by a highly competitive market, such as in central London (17). Moreover, because the original data were purchase based, we did not cover all products that were available in the market, which was a feature that could have biased our estimate of the distribution of the availability of price promotions.

Second, the current study highlighted differential responses to price promotions by social groups, with the assumption that different social groups were exposed to the same promotional environment at the national level. However, United Kingdom supermarket chains have different main target consumers and operate in different parts of the country, and hence, the promotional environment may be segmented by social groups. Our sensitivity analyses that looked at shoppers’ exposure to promotions (by taking into account the usual shopping environment for different socioeconomic groups) showed similar socioeconomic patterning in responses to promotions (Supplemental Table 4, **Supplemental Table 12**). Moreover, we did not address potential differences in purchasing across social groups within a given store.

Third, we restricted our sample to sales data from the 11 main parties of the United Kingdom grocery retail market (which account for ∼70% of the total grocery market share), thereby excluding relatively smaller grocery chains and privately owned stores. Purchasing patterns of consumers as well as marketing strategies in those stores may have been different from that of the main parties. Therefore, our findings may not be entirely generalizable.

### Implications for future research

Our measure of the frequency of price promotions provided an indication of variability of price promotions for individual products at the national level, but more-refined ways to measure the frequency of price promotions (e.g., via direct routine observations) should be developed in future research (41). Moreover, retailers and manufacturers have their own target customers. Hence, their strategies of operationalizing price promotions differ according to the social characteristics (including income and food goals) of their target consumers (15). Future studies should fully take into account the potentially different food environments provided by different retailers.

In the current study, we examined overall differences in responses to price promotions by SES groups at the population level. Future research could investigate the issue at the store level so that consumers’ responses to price promotions are analyzed within the same marketing strategy and variety of food products.

Detailed analysis of various types of price promotion (e.g., simple price reductions and multibuys) could be valuable. Recent evidence showed that restricting multibuys has failed to change the overall volume of alcohol purchased (42). However, it would be worthwhile to investigate if this finding applies to a broader set of (healthier and less-healthy) dietary categories.

Finally, the effects of price promotion on the whole food basket purchased (rather than individual products) should be evaluated. A sizeable proportion of total purchasing involved foods on promotion (Table 2), which made it at least conceivable that price promotions could have affected the overall food basket. Future analyses should involve a shopper level analysis of purchasing and diet quality (11, 43) in response to price promotion.

### Implications for policy

Our findings suggest that policies that restrict price promotions on less-healthy food categories could help achieve healthier nutrient profiles of shopping baskets for the population on average, which is likely to lead to improvements in the nutritional value of food consumed. However, we did not find evidence that restricting promotions on less-healthy versions of products within a given category would achieve a similar benefit.

In conclusion, our results imply an intriguing effect in relation to socioeconomic inequality. The SES difference in the responsiveness to promotions was more marked in healthier rather than less-healthy food categories. Moreover, the SES gap in the sales of less-healthy foods was predominantly driven by differences in off-promotion sales. Hence, the restriction of price promotions on less-healthy food categories would be unlikely to reduce the SES gap in the healthiness of food purchasing. The quest continues for measures to improve diet quality for the population as a whole while simultaneously decreasing health inequalities.

## Supplementary Material

Supplemental data
