# Supplementary material for: Price promotions on healthier compared with less healthy foods: a hierarchical regression analysis of the impact on sales and social patterning of responses to promotions in Great Britain1
Source: Am J Clin Nutr. 2015 Feb 11;101(4):808–16. doi: 10.3945/ajcn.114.094227 (PMC4381774; doi:10.3945/ajcn.114.094227)
Supplement: Supplemental data [file supp_101_4_808__index.html]

Supplemental data 

# Price promotions on healthier compared with less healthy foods: a hierarchical regression analysis of the impact on sales and social patterning of responses to promotions in Great Britain

## Supplemental data

**Files in this Data Supplement:**

- Supplemental data - Figure 1, Tables 1–12, and Text
