# Supplementary material for: Price promotions on healthier compared with less healthy foods: a hierarchical regression analysis of the impact on sales and social patterning of responses to promotions in Great Britain1
Source: Am J Clin Nutr. 2015 Feb 11;101(4):808–16. doi: 10.3945/ajcn.114.094227 (PMC4381774; doi:10.3945/ajcn.114.094227)
Supplement: Supplemental data [file 114.094227_ajcn094227SupplementaryData1.docx]

**Online Supplemental Material**

1. List of products

- Supplemental Table 1: List of food and beverage categories

1. Description of the construction of the frequency of promotions variable
2. Description of the analytic approach
3. Descriptive analysis
   - Supplemental Table 2: Sensitivity checks of Table 2
4. Results for the analysis of frequency of promotions by NP score

- Supplemental Table 3: Associations between frequency of price promotions and NP score (complete result table for Figure 1)
- Supplemental Table 4: Associations between nutrient profiling score and number of shoppers exposed to price promotions (sensitivity check of Supplemental Table 3)
- Supplemental Table 5: Associations between frequency of price promotions and NP score by food category
- Supplemental Table 6: Associations between frequency of simple price reductions and NP score
- Supplemental Table 7: Associations between frequency of multi-buys and NP score
- Supplemental Table 8: Associations between price discount rates and NP score

1. Results for the analysis of consumer responses to promotions by NP score
   - Supplemental Table 9: Association between unit sales and frequency of price promotions by nutrient profile (complete result table for Figure 2)
   - Further technical details on the transformation from the results in Supplemental Table 9 to the graphical representation in Figure 2
   - Supplemental Figure 1: Average effect and “unhealthy premium” (95% confidence interval)
   - Supplemental Table 10: Test results of differential effects by socioeconomic group in Supplemental Table 9
   - Supplemental Table 11: Association between unit sales and frequency of price promotions by NP score, separately by food category
   - Supplemental Table 12: Association between unit sales and number of shoppers exposed to price promotions by nutrient profile (sensitivity check)

**1. List of products**

**Supplemental Table 1: List of food categories**

**
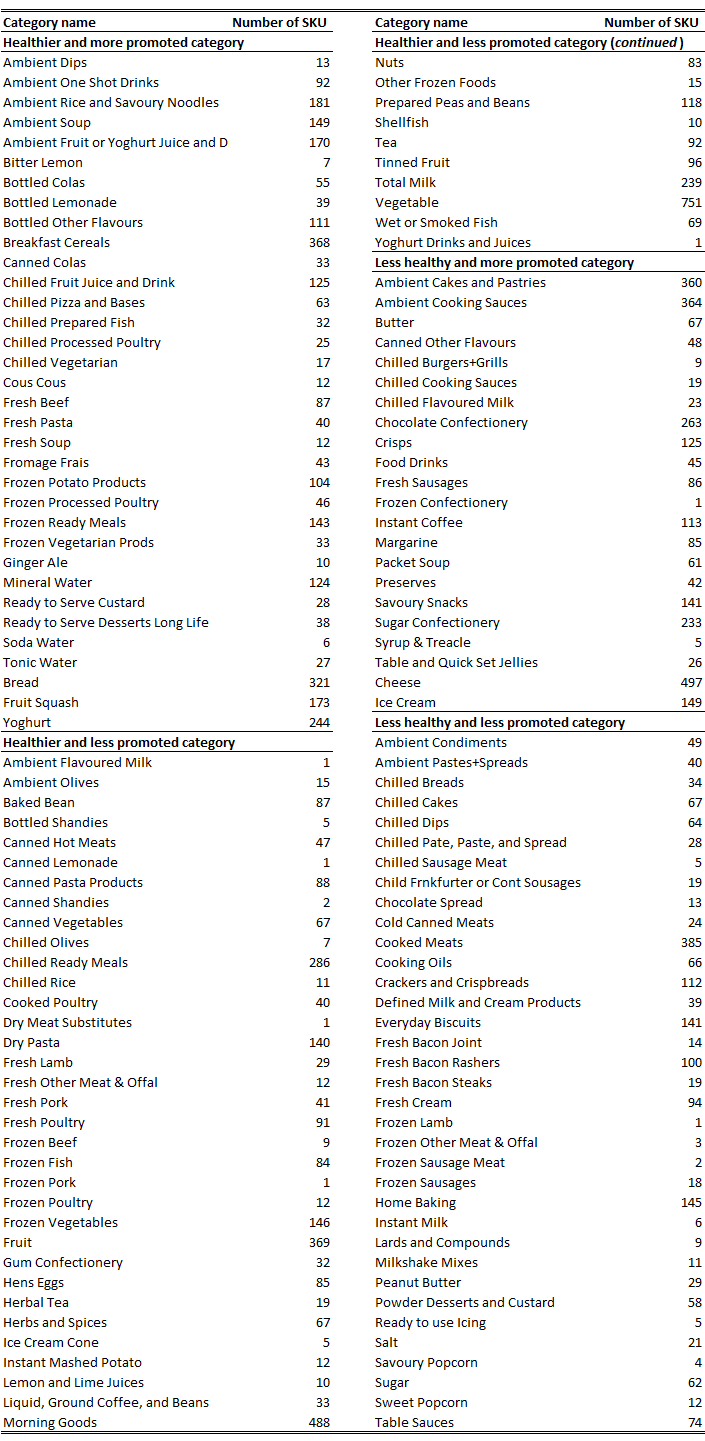
**

Note: SKU refers to Stock Keeping Unit.

**2. Construction of the ‘frequency of promotions’ variable**

**Description of the construction**

In order to assess the consumer response to price promotions, it is essential to measure which and how many price promotions were available for each product, i.e. the so-called “frequency of promotions”. However, the data researchers typically use do not contain information about price promotions, and if they contain such data, they record purchased items only. Hence, in a given store, price promotions are recorded only if an item has in fact been purchased on promotion – a feature that has thus far precluded the derivation of measures of the availability (or “supply”) of promotions. To the best of our knowledge no study has hitherto attempted to measure and include *any* measure of the supply of promotions in an analysis of sales response to price promotions at population level.

In the absence of a directly observable measure in the data, we estimated the frequency of promotions as follows: we focused on eleven “main parties” of UK multiple grocers (Tesco, Sainsbury, Asda, Morrison, M&S, Waitrose, Aldi, Lidl, Netto, Budgens, and Iceland). Those main parties are defined as such by the UK Competition Commission ([1](#_ENREF_1)). The Commission confirms that those stores follow a national pricing policy, according to which stores operate the same pricing (and thus the same price promotions) in all branches. This institutional feature offers us an opportunity to calculate how many promotions were operated in the country in a given time period: if we observe any transaction of a product that is made on promotion in a given store, it implies that the product was also on price promotion in the other branches of the same chain. It should be acknowledged, however, that to a small extent some stores still operate localised pricing in very competitive places (e.g. central London area), which is a limitation of this approach.

The frequency of promotions at a population level was constructed according to equation (S1) below: for each week *t* and for each product *j*, we counted the number of store chains *s* from which the product was purchased on promotion, while taking into account the different number of branches by store chain (n.b. different store types within the same branch, e.g. main stores and local convenience stores, were treated separately, i.e. as store chains of their own) ([2](#_ENREF_2)). The number of branches promoting each product was summed up across store chains and across the 52 weeks. Formally, the frequency of promotions (FoP) for product *j* in the year of 2010 is defined as:

$$(S1) {FoP}_{j}=\sum_{t=1}^{52} \sum_{s=1}^{11} \left( \mathbb{1}_{[{promoted}_{jts}]}\times[\# of branches s owns] \right),$$

An important assumption underlying this approach is that if a selected store chain runs a promotion on a product, at least one household that was part of the panel survey purchased the product from any branch of the same store-chain within the same week. In the data we observe more than 20,000,000 item-purchases made by 26, 986 households, distributed across the country.

Sensitivity checks were conducted using a different definition of the variable ’number of shoppers who were exposed to price promotions. The results were essentially identical to those obtained from using the above proxy (for more details see Supplemental Table 8 and 12).

**3. Description of the analytic approach**

A hierarchical regression model was employed. For item *j* in category *c*, the following base model was specified:

$$\log\left( {FoP}_{jc} \right)=\beta_{0c}+\beta_{1c}\mathrm{NP}_{jc}+e_{jc}.$$

The term $NP$ represents the nutrient profile. The term $e_{jc}$ is the idiosyncratic error. This basic estimation tells us whether less healthy items are more frequently promoted than healthier ones. We further specify that the baseline frequency of promotions (intercept: $\beta_{0c}$) and the association with the NP score (slope: $\beta_{1c}$) vary by dietary category and that this variation is a function of genuine healthiness of each dietary category:

$$\beta_{0c}=\gamma_{00}+\gamma_{01}\bar{\mathrm{NP}_{c}}+u_{0c},$$

$$\beta_{1c}=\gamma_{10}+\gamma_{11}\bar{\mathrm{NP}_{c}}+u_{1c}.$$

$\bar{\mathrm{NP}_{c}}$ is the mean Nutrition Profile score of products in category *c*. Substituting the above two equations into the base equation yields another reduced-form equation:

$$Eq\left( 1 \right): \log\left( \mathrm{FoP}_{jc} \right)=\gamma_{00}+\gamma_{01}\bar{\mathrm{NP}_{c}}+\gamma_{10}\mathrm{NP}_{jc}+\gamma_{11}\left[ {\bar{\mathrm{NP}_{c}}\times\mathrm{NP}}_{jc} \right]+\left\{ u_{0c}+u_{1c}\mathrm{NP}_{jc}+e_{jc} \right\}.$$

Note that $\mathrm{NP}_{jc}$ is centred at $\bar{\mathrm{NP}_{c}}$, and therefore the coefficient of $\mathrm{NP}_{jc}$ captures the variation of NP score from the mean of the category (i.e. within-category effect), and $\bar{\mathrm{NP}_{c}}$ captures the between-category effect of healthiness. The interaction term indicates whether a less healthy version of the product in a less healthy category is more frequently promoted than a less healthy version of the product in a healthier category.

*Differential consumer responses to promotions by healthiness*

In order to address Question 2 (“Are consumers more responsive to promotions on less healthy products?”), we investigate the differential effects of frequency of promotions by NP score of products. The baseline product-level purchases equation is given by:

$$\log\left( \mathrm{Sales}_{jc} \right)=\beta_{0c}+\beta_{1c}\log\left( \mathrm{FoP}_{jc} \right)+\beta_{2c}\mathrm{NP}_{jc}+\beta_{12c}\left[ \log\left( \mathrm{FoP}_{jc} \right)\times\mathrm{NP}_{jc} \right]+\boldsymbol{Z}_{jc}^{\mathbf{'}}\boldsymbol{\delta}+e_{jc}.$$

The outcome variable is the log of total number of products *j* in category *c* that were purchased over 52 weeks. The interaction term $\left[ log(\mathrm{FoP}_{jc})\times\mathrm{NP}_{jc} \right]$ measures whether, and if so, to what extent the effect of promotions varies by healthiness of the product. The vector $\boldsymbol{Z}_{jc}$ includes a set of covariates known to affect sales, including the reference price, average rate of price discount when promoted, and indicators of brands (which captures brand-specific feature of each product) ([3](#_ENREF_3)).

Similarly to the previous analysis, category-specific coefficients are modelled as follows:

$$\beta_{0c}=\gamma_{00}+\gamma_{01}\bar{\mathrm{NP}_{c}}+u_{0c},$$

$$\beta_{1c}=\gamma_{10}+\gamma_{11}\bar{\mathrm{NP}_{c}}+u_{1c},$$

$$\beta_{2c}=\gamma_{20}+\gamma_{21}\bar{\mathrm{NP}_{c}}+u_{2c},$$

$$\beta_{12c}=\gamma_{30}+\gamma_{31}\bar{\mathrm{NP}_{c}}+u_{3c}.$$

Substituting these four equations into the original equation we obtain the following reduced-form:

$$Eq\left( 2 \right):\log\left( \mathrm{Sales}_{jc} \right)=\gamma_{00}+\gamma_{10}\log\left( \mathrm{FoP}_{jc} \right) +\gamma_{01}\bar{\mathrm{NP}_{c}}+\gamma_{20}\mathrm{NP}_{jc}+\gamma_{21}\left[ \bar{\mathrm{NP}_{c}}\times\mathrm{NP}_{jc} \right] +\gamma_{11}\left[ \log\left( \mathrm{FoP}_{jc} \right)\times\bar{\mathrm{NP}_{c}} \right] +\gamma_{30}\left[ \log\left( \mathrm{FoP}_{jc} \right)\times\mathrm{NP}_{jc} \right] +\gamma_{31}\left[ \log\left( \mathrm{FoP}_{jc} \right)\times\bar{\mathrm{NP}_{c}}\times\mathrm{NP}_{jc} \right]+\boldsymbol{Z}_{jc}^{\mathbf{'}}\boldsymbol{\delta}+\left\{ u_{0c}+u_{1c}\log\left( \mathrm{FoP}_{jc} \right)+u_{2c}\mathrm{NP}_{jc}+u_{3c}\left[ \log\left( \mathrm{FoP}_{jc} \right)\times\mathrm{NP}_{jc} \right]+e_{jc} \right\}.$$

As in the previous model, $\mathrm{NP}_{jc}$ is centred at $\bar{\mathrm{NP}_{c}}$ to distinguish the effect of within-category variations of NP score from the effect of between-group variations. The interaction term $\left[ log(\mathrm{FoP}_{jc})\times\bar{\mathrm{NP}_{c}} \right]$ captures whether consumers are more responsive to a promotion in the less healthy food category, compared to a promotion in a healthier category, i.e. between-category effect. On the other hand, the term $\left[ log(\mathrm{FoP}_{jc})\times\mathrm{NP}_{jc} \right]$ indicates whether consumers are more responsive to promotion on a less healthy version of the product, compared to a healthier version within the same category, i.e. within-category effect. The triple interaction term $\left[ log(\mathrm{FoP}_{jc})\times\bar{\mathrm{NP}_{c}}\times\mathrm{NP}_{jc} \right]$ captures the cross effects of the above interaction terms.

All models were estimated via a restricted maximum likelihood technique. Stata MP Version 12 was used.

**4. Descriptive analysis**

**Supplemental Table 2: Sensitivity checks of Table 2 in the main text**

In this table, food categories scoring 0 or higher in the Nutrient Profile score, and beverages scoring -3 or higher are grouped as a ‘Less Healthy category’, and otherwise as a ‘Healthier category’.

Notes: See notes of Table 2. SES group differences: In each column, the mean numbers *not* sharing a symbol * or † are significantly different from each other at 5% level, based on two-sided T-tests. The tests were Bonferroni corrected.

**5. Results for the analysis of frequency of promotions by NP score**

**Supplemental Table 3: Associations between frequency of price promotions and nutrient profiling score (product-level, category-level, and the interaction) (Table related to Figure 1)**

**
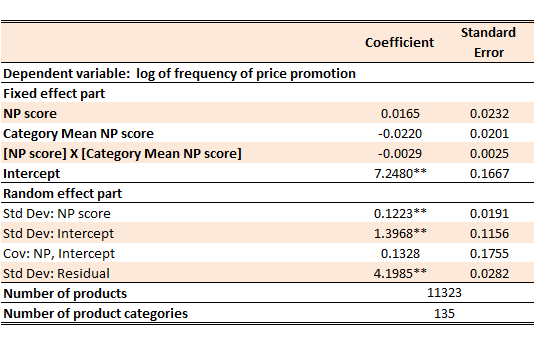
**

Notes: **: p<0.01, *: p<0.05.

**Supplemental Table 4: Associations between nutrient profiling score and number of shoppers exposed to price promotions (sensitivity check, see section 2 of this document)**

**
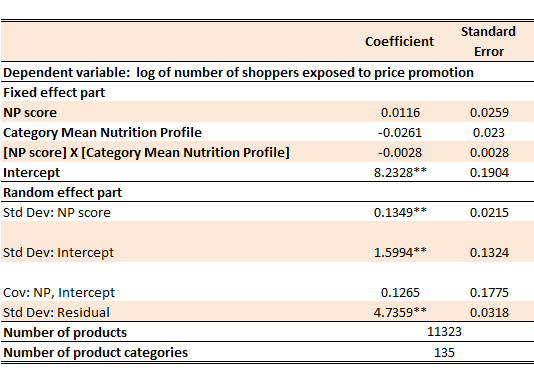
**

**Notes:** **: p<0.01, *: p<0.05. Results in this table represent a sensitivity check of the variable for promotion environment – “the number of shoppers who were exposed to price promotions.”

**Supplemental Table 5: Associations between frequency of price promotions and NP score by food category Coefficient of nutrition profile. Dependent variable: log of frequency of price promotions.**

**
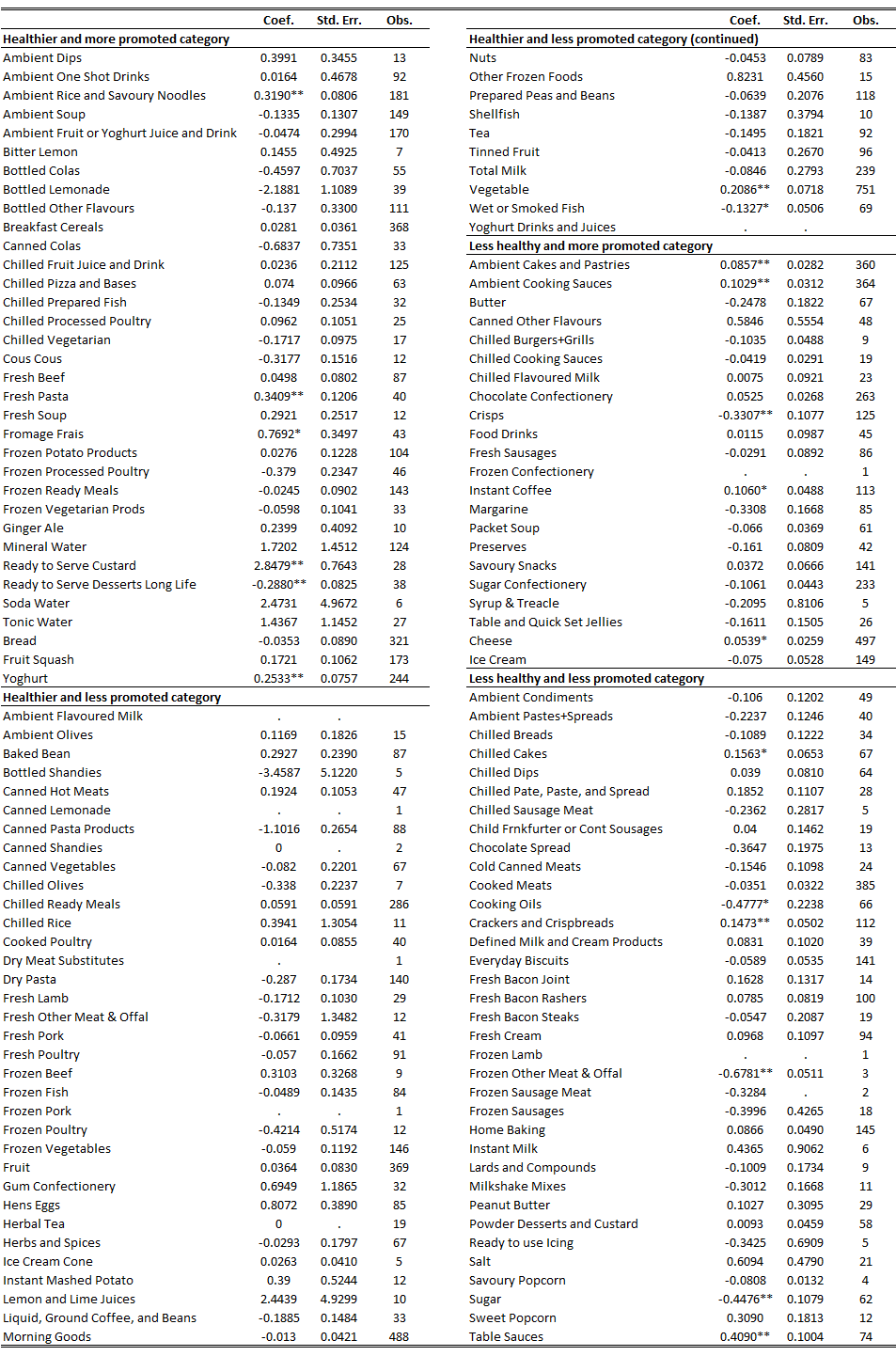
**

Notes: **: p<0.01, *: p<0.05. We estimated the following base model for each food category separately: for item j,$\log\left( {FoP}_{j} \right)=\beta_{0c}+\beta_{1}\mathrm{NP}_{j}+e_{j}$. Note that ${FoP}_{j}$ and $\mathrm{NP}_{j}$represent the frequency of price promotions and nutrition profiling score of item j, respectively. In this table, the estimated coefficient $\beta_{1}$ and its standard error are reported.

**Supplemental Table 6: Associations between frequency of simple price reductions and NP score**

**
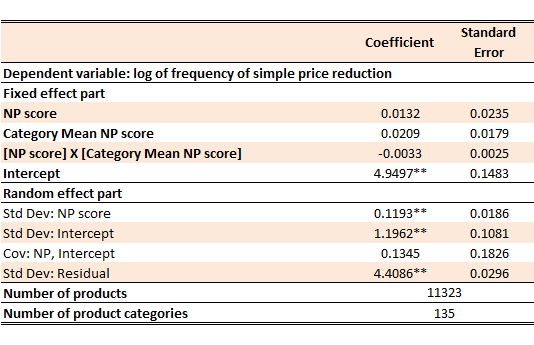
**

Notes: **: p<0.01, *: p<0.05.

**Supplemental Table 7: Associations between frequency of multi-buys and NP score**

**
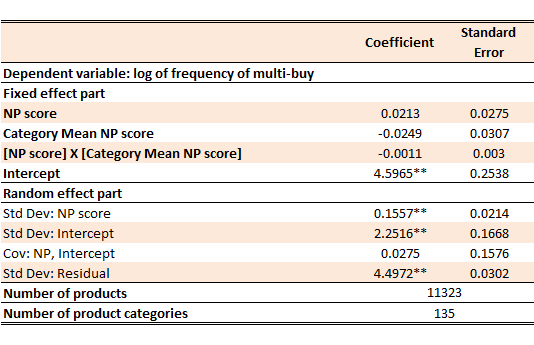
**

Notes: **: p<0.01, *: p<0.05.

**Supplemental Table 8: Associations between price discount rates and NP score**


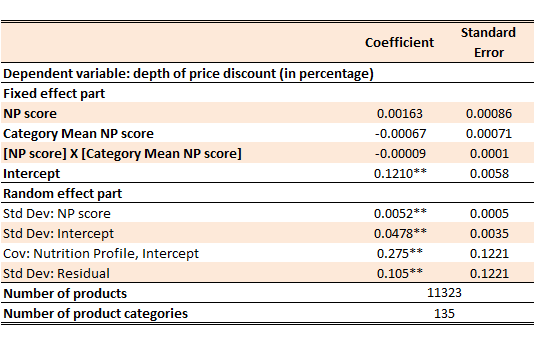


Notes: **: p<0.01, *: p<0.05.

**6. Results for the analysis of consumer responses to promotions by NP score**

**Supplemental Table 9: Association between unit sales and frequency of price promotions by nutrient profile (product-level, category-level, and the interaction), overall and by socioeconomic groups. Dependent variable: log of annual unit sales. (Table related to Figure 2)**

Notes: **: p<0.01, *: p<0.05. The analysis is based on a mixed effect model. Fixed effect coefficients only are presented for brevity (full results including random effect coefficients are available upon request). Other covariates in the model include: reference price, average rate of price discount (through the survey year) when price was promoted, dummy variables indicating brands (which capture brand-specific features of products). Column (1) gives the results for unit sales that are calculated from the purchases of the whole population in the sample. Column (2), (3), and (4) represent the subsample analyses, in which the unit sales made by each socioeconomic group separately are used.

**Further technical details on the transformation from the results in Supplemental Table 9 to the graphical representation in Figure 2**

This subsection explains how to transform the results in Supplemental Table 9 to Figure 2 in the main paper.

As explained in the note to Figure 2, the bars in Figure 2 comprise two coefficients presented in Supplemental Table 9: (a) the coefficient of “log(Frequency of promotions)”; and (b) the coefficient of “[log(Frequency of promotions) X [Category mean NP score].” Note that the coefficient of “log(Frequency of promotions)” in Supplemental Table 9 presents an increase of sales when, all else equal, the frequency of price promotions is increased by 1%. In order to examine a more intuitive scenario, in Figure 2, we calculated the effect when the frequency of promotions is increased by 10% by simply multiplying the coefficient by 10 (see Notes of Figure 2 in the main text).

Next, the coefficient of “[log(Frequency of promotions) X [Category mean NP score]” in Supplemental Table 9 gives an additional effect of promotions when category-level NP score is increased by 1 point. In Figure 2, we calculated the effect when the category-level NP score is increased by 1 standard-deviation (SD) point. As presented in the main paper, the SD of category-level NP score was 6.96.

In the estimation using all households, a SD point increase in the category-level NP score (implying that the food category becomes less healthy) was associated with an additional 7.66 (=1.1 X 6.96) percentage point increase of the effect (p<0.01). Note that, since we calculated the effect of increasing promotions by 10%, the coefficient of the interaction term was also multiplied by 10, i.e., 0.0011 x 10=1.1%. This part of sales uplift is presented by “unhealthy premium” (red area) in **Supplemental Figure 1** below (“All” column, 7.7%).

**Supplemental Figure 1: Average effect and “unhealthy premium” (95% confidence interval)**


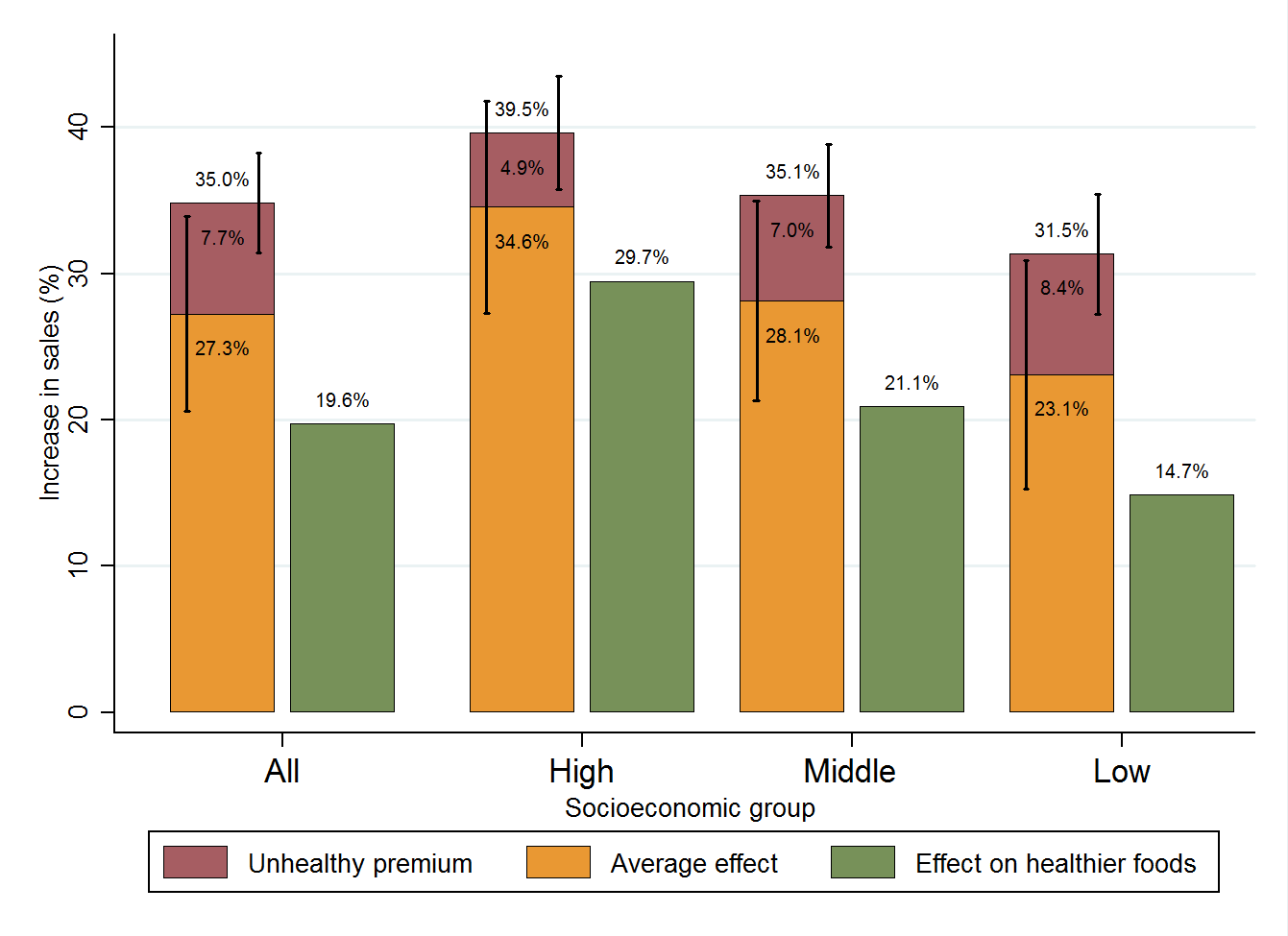


The effect on less healthy food category is represented by the average effect plus the “unhealthy premium” (red and orange area in Supplemental Figure 1). On the other hand, the green area shows the effects in healthier category (the average effect minus the “unhealthy premium”).

The same calculations were conducted for the SES subsamples. The “unhealthy premiums” are statistically significant for all socioeconomic groups (p<0.01 for all groups, see Supplemental Table 9). Note that, between groups, the size of the “unhealthy premium” were significantly different between the highest and the lowest group (p<0.01); were marginally significantly different between the highest and the middle group (p=0.12); and were not statistically different between the middle and the lowest group (p=0.54) (Supplemental Table 10).

The “within-category” effects were statistically indistinguishable from zero for all groups (the coefficients of “[log(Frequency of promotions) X [NP score]” in Supplemental Table 9), and therefore were not visualised.

**Supplemental Table 10: Test results of differential effects by socioeconomic group in Supplemental Table 9**


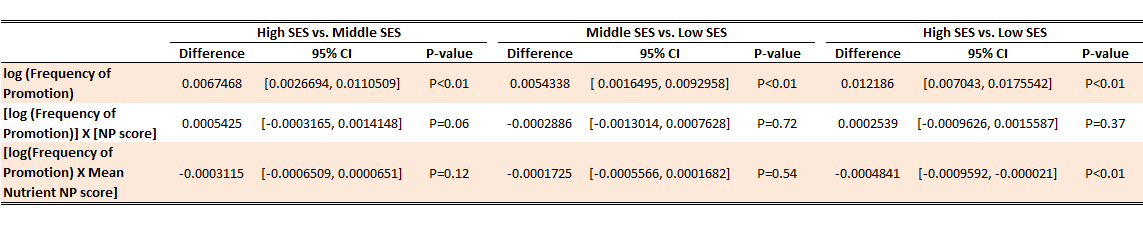


Notes: The results were based on bootstrap two-sided tests (1,100 times replications). 95% confidence intervals of the differences are presented. First, the regression coefficients were estimated using bootstrapped data for each SES group independently. Then, we calculated the difference in the coefficients between SES groups and also the 95% confidence intervals. Finally, we tested whether or not the difference in the coefficients between SES groups was statistically zero.

**Supplemental Table 11: Association between unit sales and frequency of price promotions by NP score, separately by food category. Coefficient of log (Frequency of Promotions)] X [NP score]. Dependent variable: log of unit sales**


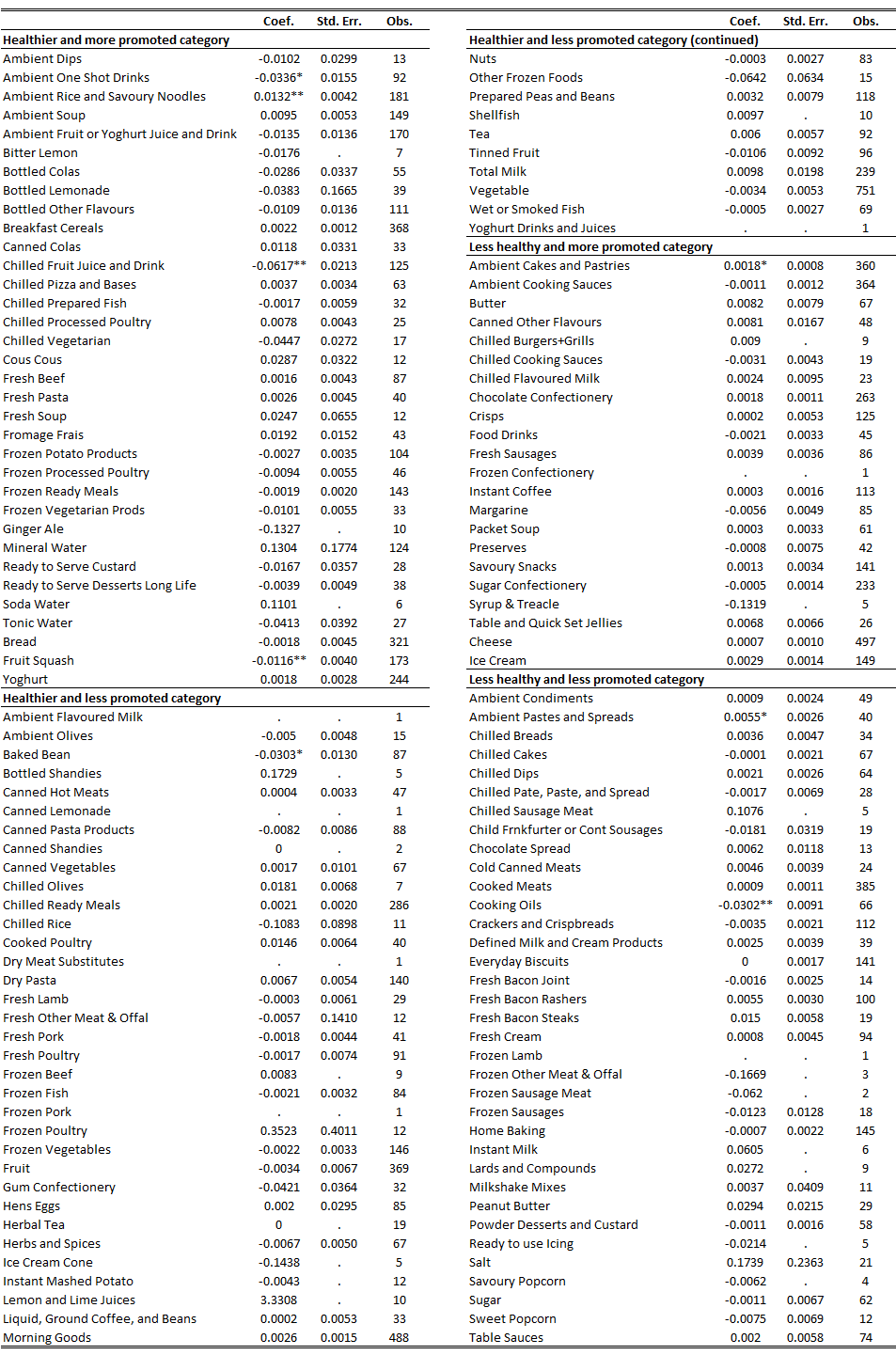


Notes: **: p<0.01, *: p<0.05. We estimated the following base model for each food category separately. For item j, $\log\left( \mathrm{Sales}_{j} \right)=\beta_{0}+\beta_{1}\log\left( \mathrm{FoP}_{j} \right)+\beta_{2}\mathrm{NP}_{j}+\beta_{12}\left[ \log\left( \mathrm{FoP}_{j} \right)\times\mathrm{NP}_{j} \right]+\boldsymbol{Z}_{j}^{\mathbf{'}}\boldsymbol{\delta}+e_{j}$. Note that ${FoP}_{j}$ and $\mathrm{NP}_{j}$represent the frequency of price promotions and nutrition profiling score of item j, respectively. The vector $\boldsymbol{Z}_{j}$ includes the reference price, average rate of price discount when promoted, and indicators of brands. In this table, the estimated coefficient $\beta_{12}$and its standard error are reported. This coefficient gives the interaction between the frequency of price promotions and nutrient profile, indicating the degree to which the association between sales and price promotions differs by nutrient profile.

**Supplemental Table 12: Association between unit sales and number of shoppers exposed to price promotions by nutrient profile (sensitivity check, see section 2 of this document)**

**
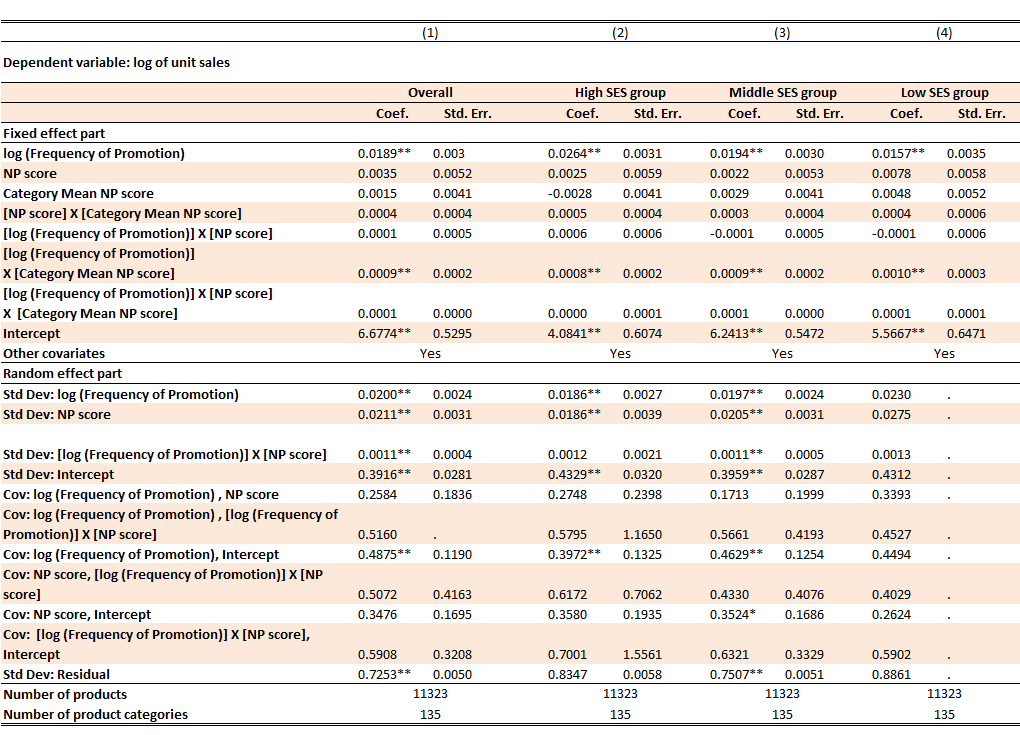
**

Notes: See notes of Supplemental Table 9. Sensitivity check of the variable for promotion environment – “the number of shoppers who were exposed to price promotions.” In the random effect part of the analysis for low SES group, the likelihood function did not converge.

**References**

1. Competition Commission. Supermarkets, a report on the supply of groceries from multiple stores in the United Kingdom, Volume 2: Background chapters. In: Office S, editor. London2000.

2. IGD Retail Analysis. Datacentre. URL: <http://retailanalysis.igd.com/Hub.aspx?id=9&tid=7> Accessed January 2013.

3. Bray JW, Loomis B, Engelen M. Correlates of in-store promotions for beer: differential effects of market and product characteristics. Journal of studies on alcohol and drugs. 2007;68(2):220-7.

4. Starfield B. Pathways of influence on equity in health. Social Science & Medicine. 2007;64(7):1355-62.

5. Mishra A, Mishra H. The influence of price discount versus bonus pack on the preference for virtue and vice foods. Journal of Marketing Research. 2011;48(1):196-206.
